# Development of a novel virtual reality-enabled remote monitoring device for maintenance of cathodic protection systems on oil and gas pipelines

**DOI:** 10.1038/s41598-023-43159-x

**Published:** 2023-09-23

**Authors:** Chika Edith Mgbemena, David Obike Onuoha, Harold Chukwuemeka Godwin

**Affiliations:** Department of Industrial/Production Engineering, Nnamdi Azikiwe University, P.M.B. 5025, Awka, Anambra Nigeria

**Keywords:** Engineering, Materials science

## Abstract

Periodic inspections are required for oil and gas pipelines equipped with cathodic protection systems (CPS) to prevent corrosion. This inspection takes time and requires money, mobilisation of specialised staff, and accessibility of pipeline right of way which is often hindered by security issues, and bad terrain. A novel virtual reality-enabled remote monitoring device, developed using the NACE SP0169 standard, which measures the pipe-to-soil potential (PSP) reading, transmits the measured data to an IoT server, and a virtual environment for analysis and feedback while providing an immersive experience to the user, all in real-time, is presented. The hardware and software components are developed using Sensors, Arduino Mega 2560 board, AWS Sumerian 1.2.0 software, ThingSpeak IoT server, Blender modelling software and AutoCAD. When implemented on real test posts of oil and gas facilities and compared with the traditional methodology, the system showed consistency of data measurement and analysis, with real-time feedback to users, and a Spearman rank’s correlation coefficient of 0.998944. The study is significant as it provides the oil industry with an expert, easy-to-understand tool which helps to reduce the rate of oil spillage and losses through corrosion.

## Introduction

Pipelines lose longevity due to corrosion^1^. To evaluate pipeline performance and detect flaws early on for preventive maintenance of facilities, corrosion monitoring is essential^2^. This is because losses can be minimised through early prediction and effective corrosion monitoring^3^.

Corrosion is an occurrence that has negative financial effects on some sectors ^4^. According to the National Association of Corrosion Engineers (NACE) and the World Corrosion Organisation (WCO), the yearly cost of corrosion-related issues worldwide is estimated to be $US2.4 trillion, or 3% of global GDP^2,3^. Over 7359 spills with a total release of 3,114,255 barrels, valued at $247,957,000, have occurred in Nigeria as of 2012. This amounts to the greatest rate of spills globally, with an average of nearly 600 each year^5^. This spillage largely results from continuously checking the condition of these structures and communicating pipeline failure due to corrosion. Corrosion was the primary cause of the oil pipeline explosion in Qingdao, in the eastern Shandong province of China, in 2013, as it weakened the pipeline’s wall, resulting in the break and incurring a significant economic loss of $US123.9 million^2^. Internal and external corrosions affect the security and integrity of pipelines over time; thus, pipelines always need continuous inspection^6^.

Due to the COVID-19 pandemic’s devastating effects on the oil and gas industries, it is imperative to adopt automation and digitalisation to increase production resilience^7^.

One of the most popular oil and gas sector techniques to stop or reduce rust and corrosion of structures and metal pipelines is cathodic protection (CP)^8–10^. CPS are fundamental to pipeline integrity management, and the structural complexity of the pipeline networks makes its maintenance challenging^11^. CPS are installed in oil and gas pipelines to protect them from rust and corrosion. To determine the pipeline’s protection level against corrosion, stray currents, and localised coating defects, specialised personnel must conduct periodic inspections, data capture and data analysis. Traditionally, the popular tool used to measure the PSP readings on test points is the Multimeter. The obstacles involved in conducting this routine inspection using the traditional methodology are their length, expense, need for skilled staff, and accessibility to the pipeline right of way^12^. Oil and gas facilities are in significant danger of corrosion when the CPS is not adequately protecting them because the pipelines are at high risk of corrosion-related problems which can result in pipeline leakages leading to environmental pollution, high cost of maintenance, and consequently, losses, legal battles, and even death^2^. This research aims to address the difficulties brought on by circumstances such as lockdowns imposed during the Covid-19 pandemic outbreak, security/community concerns that impose movement restrictions, and inaccessibility of pipeline right-of-way, which frequently need time and money to fix. Hence, it is important to inform the facility owners about the degree of protection provided against corrosion and potential current leaks. This makes remote monitoring essential, to continuously check the condition of these structures and communicate the information to users in real-time^13^. Remote monitoring is a new development that automates the data collection process and provides operators with a proactive surveillance system. A remote corrosion monitoring system consists of various sensors, a power supply, a data acquisition system and a cellular network for data transfer. Solar panels can be used in the absence of electrical power grids to supply power. Due to the long-distance and widespread distribution of pipelines, remote monitoring of pipelines solves the problems associated with the manual measurement method. It is suitable for inaccessible locations, which may be due to distance, hostile environments, heights, or severe operating conditions^13^. It also guarantees automatic, repeatable, and reliable results. The system generates high-quality data in an easy-to-interpret manner without the need for an expert. The application of remote monitoring reduces operational monitoring costs.

The use of sensors and microcontrollers has been recommended as a low-cost technology for corrosion control in pipelines^2^. Various industry 4.0 technologies such as data analytics, the Internet of Things (IoT) and the use of machine learning techniques that result in smart methods^14,15^, have successfully been implemented for the protection of pipelines^9,16–18^. To speed up CPS maintenance, it is necessary to identify early danger signs and notify operators so they can take quick action. A CPS voltage monitoring device which captures PSP readings with an accuracy of 96.2% to 98.8% has been developed using an integrated op-amp circuit, a NEO-6M GPS Module and a Blynk analytics platform^19^.

Monitoring the CPS of buried Pipelines against corrosion has been implemented using a potential probe^20^, a potential array of sensors which receive, store, and analyse data from the monitoring node measurement system^21^, and by using non-intrusive ultrasonic sensors^22^. A monitoring unit which is based on fuzzy logic and an existing wM-Bus at 169-MHz infrastructures for gas metering has been developed by Ref.^23^ for use in the monitoring and control of pipelines against Corrosion.

Intelligent monitoring systems that employ standard communication technologies such as cellular phones, antennas and GPS devices to ensure continuous monitoring have also been employed in CPS monitoring^24–26^. A CPS monitoring unit that analyses the potential difference with zero voltage divider error has been developed by Ref.^12^ using an ADS1115 voltage sensor and Arduino-based measurement unit. A supervisory control and data acquisition (SCADA)-based system has been used to monitor and control the CPS on pipelines^26,27^. The use of wireless sensor network technology (WSN) that captures potential data and remotely transmit it to appropriate quarters has been implemented for remote monitoring of CPS^28,29^. Many detection systems deployed for monitoring in oil and gas industries are based on WSN systems or SCADA systems with limitations. WSN-based systems are incompatible and not homogenous systems. They lack coordinated communication and transparency among regions and processes, while SCADA systems are expensive, inflexible, not scalable, and provide data with a long delay^30^.

Virtual Reality (VR) technology enables problems to be predicted, solved, and controlled by connecting real sites with virtual ones in real-time^31^. The immersive experience of VR systems has been applied in various real-time simulated events involving sensors which transmit captured data to cloud servers for easy visualisation^32–35^.

While several works have been reported in the literature on the techniques used to monitor the CPS on pipelines against corrosion, none have been reported to employ VR technologies to achieve an easier and more effective process. This gap was addressed in this research.

Table 1 shows a summary of the comparative analysis of the characteristics of devices found in literature and that of the proposed work, thus demonstrating the difference between the works.

**Table 1:** Differences between the devices found in literature and that of the proposed work.

Characteristics	Existing and proposed work	
	Existing work	Proposed work
Manual measurements requiring specialised staff on site	✓	X
Offline data analysis	✓	X
Real-time feedback	✓	✓
IoT-enabled	✓	✓
High-quality data generated	✓	✓
Remote monitoring of pipelines	✓	✓
Compatibility with existing facilities	X	✓
Delayed feedback	✓	X
Real-time data analysis	✓	✓
Easier and more effective visualisation of data	X	✓
Provides an immersive experience to the user	X	✓

Hence, the proposed work aims to design and implement an intelligent VR-enabled system that captures and analyses the PSP readings of the CPS installed in buried pipelines, while providing real-time visual and audio feedback to the users, with an immersive experience. The proposed system will be achieved using the Internet of Things, VR, cloud computing and data analytics technologies. This paper, therefore, presents an intelligent system that consists of a remote monitoring device installed at the CPS test post to measure the PSP readings, analyse the data, transmit it to a web server and a virtual environment, and alert the user when the CPS is experiencing any problems that may need immediate intervention. The study contributes significantly to the oil and gas industry as it provides it with an expert, easy-to-understand system which helps to reduce the rate of oil spillage and losses through corrosion.

To assess the performance and effectiveness of the device, some evaluation indicators are considered and incorporated. The selected indicators are summarised in Table 2.

**Table 2:** Evaluation Indicators for assessing the performance and effectiveness of the device.

S/N	Evaluation indicators	Implementation plan
1	Industry standards, guidelines, and regulatory requirements	The proposed device will be developed using the NACE SP0169 standard
2	Accuracy	The accuracy of the proposed device will be confirmed by comparing its readings with that of the conventional multimeter
3	Communication range	The proposed device will be configured to be compatible with any available mobile network
4	Data logging and reporting	Data is logged in the ThingSpeak IoT platform and are easily transmitted into the Virtual Reality environment
5	Remote monitoring	The device’s remote monitoring capabilities will have features like real-time monitoring, alerts or notifications for critical events or deviations from desired values and an immersive experience when viewed with a VR headset
6	Power efficiency	The proposed device will be equipped with a good battery that can last for up to 12 h and be recharged with a renewable energy source

## Methods

The methods involved in the development of the VR-enabled CPS monitoring system include the development of the hardware and software components of the system, the development of the CAD representation of the actual pipeline using AutoCAD, modelling of the CP components using the Blender 3D modelling software, modelling of the virtual environment using the AWS Sumerian cloud computing software, selection of appropriate VR headset, synchronisation, deployment and validation.

### Design of the hardware component of the CPS remote monitoring device

Due to its inexpensive cost and availability of free hardware training software, the Arduino hardware microcontroller was selected. It is compatible with a variety of sensors and parts that could be used in research. The measuring range of the proposed system is configured to encompass 0 to 5 V because CPS monitoring readings are often less than 2.5 V, and the Arduino Mega 2560 board was chosen because it can give up to 5 V and have more inputs and outputs (I/O) which can accommodate more peripherals when needed.

The ThingSpeak IoT Platform was selected based on low-cost, and little or no server setup, for data collection from the CPS Remote Monitoring device and feedback to the users.

The GPRS mode of transmission is selected for the data transmission to the IoT server due to its capabilities which include low installation costs, ease of installation, and ease of data measurement. Hence, the SIM900 GSM/GPRS Shield with a Sim was selected. Two 9 V batteries were selected to power the device. Connections are made using jumper cables.

**Figure 1:**
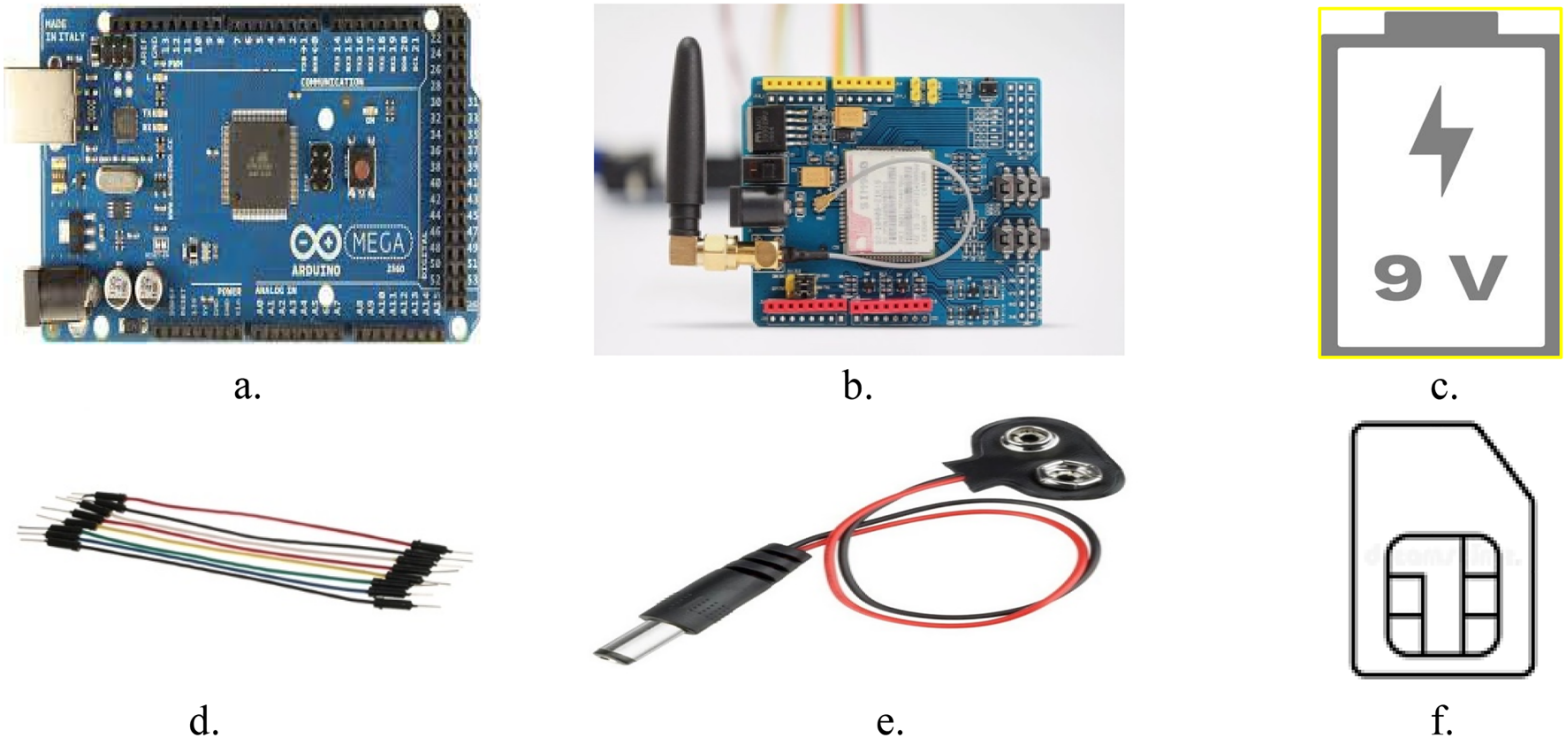
CPS remote monitoring device components (**a**) Arduino Mega 2560. (**b**) SIM900 GSM/GPRS Shield. (**c**) 9 V battery. (**d**) Jumper cable. (**e**) 9 V battery connector with barrel jack. (**f**) Simcard.

The components used for the development of the hardware component of the CPS Remote monitoring device are depicted in Fig. 1.

The steps taken to assemble and build the device are described thus:

1. Jumper cable is connected from TX1 (pin 18) of Arduino Mega 2560 to D1 of SIM900 GSM/GPRS Shield for transmitting data from Arduino Mega 2560 to SIM900 GSM/GPRS Shield.
2. Jumper cable is connected from RX1 (pin 19) of Arduino Mega 2560 to D0 of SIM900 GSM/GPRS Shield for receiving data from SIM900 GSM/GPRS Shield to Arduino Mega 2560.
3. Jumper cable is connected from the GND of Arduino Mega 2560 to the GND of SIM900 GSM/GPRS Shield for grounding of the SIM900 GSM/GPRS Shield.
4. Jumper cable is connected to the A0 (Analog Pin) of Arduino Mega 2560 while the other end is open. Terminal 1 for taking PSP measurement which connects to the reference electrode cable terminated at the test post.
5. Jumper cable is connected to the GND of Arduino Mega 2560 while the other end is open. Terminal 2 for taking PSP Measurement which connects to the test cable from pipeline terminated at the test post.
6. 9 V Battery is connected to the Arduino Mega 2560 using a barrel jack to power the Arduino Mega 2560.
7. 9 V Battery is connected to the SIM900 GSM/GPRS Shield using a barrel jack to power the SIM900 GSM/GPRS Shield.
8. Sim card is inserted into the sim track of the SIM900 GSM/GPRS Shield for a GPRS internet connection.
9. The connected components are then placed inside a casing as shown in Fig. 5, for protection of the components during usage.

**Figure 2:**
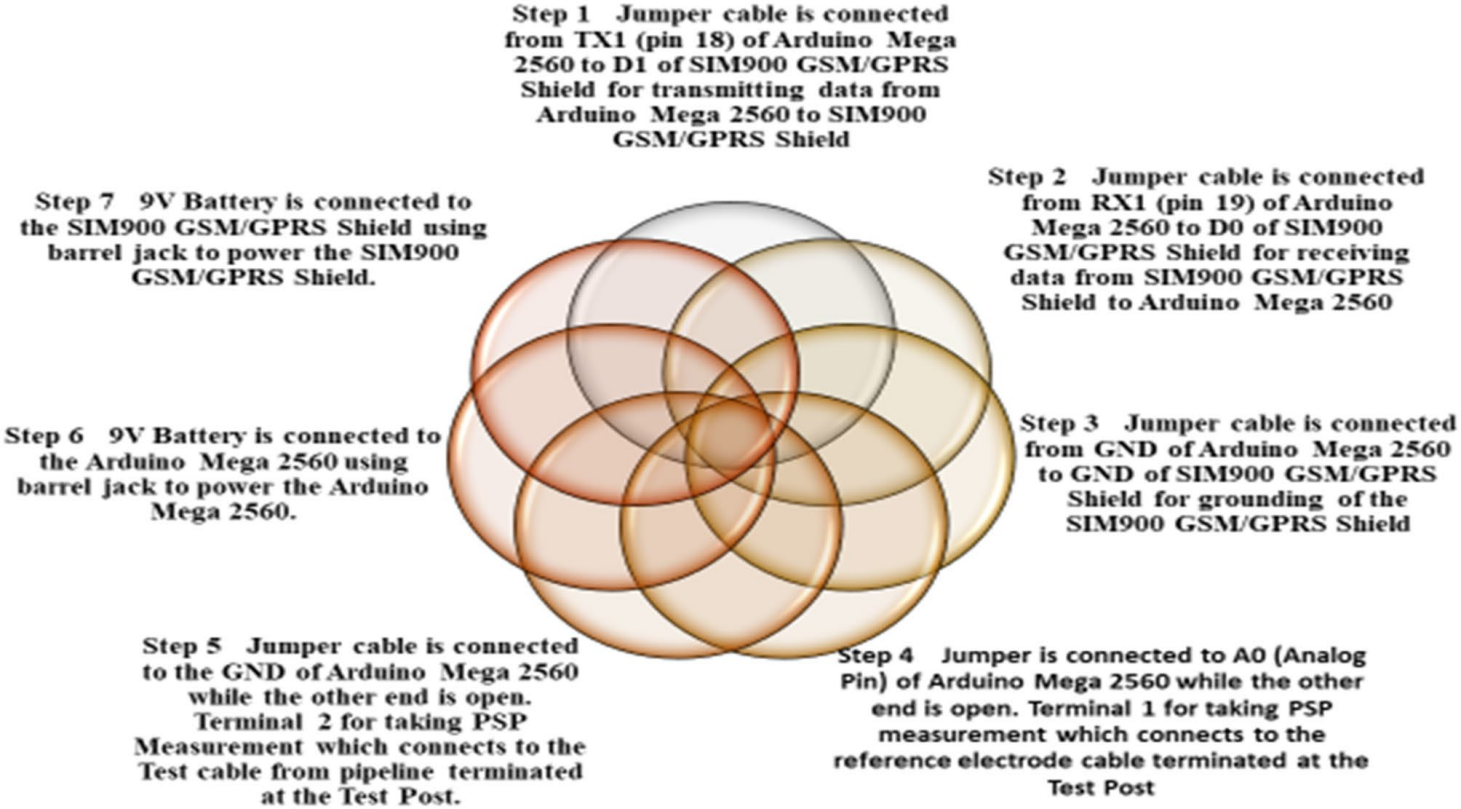
Steps taken to assemble and build the device.

These steps are summarised in Fig. 2.

### Development of the software component of the CPS remote monitoring device

Lines of codes are written to measure data between test cables and reference electrode cables, analyse the data, check for the GPRS network, establish connections with the ThingSpeak IoT server, transmit the captured PSP readings to the ThingSpeak IoT servers, close the connections and repeat the process. These codes are written using the programming language provided by Arduino Software.

### Modelling the VR environment

The CAD representation of the actual pipeline with test post under the CP system was developed using AutoCAD software. The 3D modelling of the CP system components required for visualisation in the VR environment was modelled using the Blender modelling software. Blender was chosen for the modelling due to its low-cost, great functionalities, and the ability to export its models in various formats that work with a variety of VR modelling programs. The test post, the cables from the pipeline, and the reference electrode is the primary component that is modelled.

The VR environment is modelled using the AWS Sumerian cloud computing software, with designs from the CAD software imported into the AWS Sumerian. The 3D models designed using blender software are exported in film box (.fbx) file format which are formats that can easily be imported into the AWS Sumerian 1.2.0.

### Simulation

The model created is simulated using the Java Script, State machine and the Animation features of the AWS Sumerian.

### Visualisation

A VR headset was selected after modelling the VR environment and importing component designs from the 3D software into the VR model. Considerations for selecting the VR headset include the field of view, display frequency, tracking system, types of controllers, and weight and ergonomics. The VR Glass headset is selected due to its low cost and compatibility.

### Synchronisation

The ThingSpeak IoT platform was used to synchronise the VR environment with the real-time data from the developed device. The data from the ThingSpeak IoT server is configured to be collected and analysed by the VR model in AWS Sumerian, which is constantly being updated. This will be accomplished by combining AWS Sumerian’s state machine, Script, and HTML elements.

### Deployment

The VR Model published on the AWS Sumerian web server is deployed to mobile devices and computers.

### Setting the limits of the PSP readings for analysis and feedback

The system utilises the knowledge from the National Association of Corrosion Engineers (NACE) international SP0169 standard, to analyse the PSP data and provide real-time feedback. This criterion states that adequate protection is achieved if the value is at least a negative potential of − 0.85 V, concerning the copper/copper sulphate electrode contacting the electrolyte. This criterion was used to determine the upper and lower limits of the potential reading. Hence, PSP values of − 1.3 Vcse and − 0.850 Vcse were chosen for the upper and lower limits respectively.

### Test and validation

The developed system is tested at the facility of the Integrated Corrosion Science Company Limited (ICSCL), an oil and gas contractor located in Port Harcourt, Nigeria. ICSCL, which specialises in corrosion control and monitoring, deployed their CP Technician and operator to the readings and monitor the data. The company’s consent was obtained in written form before the tests.

**Figure 3:**
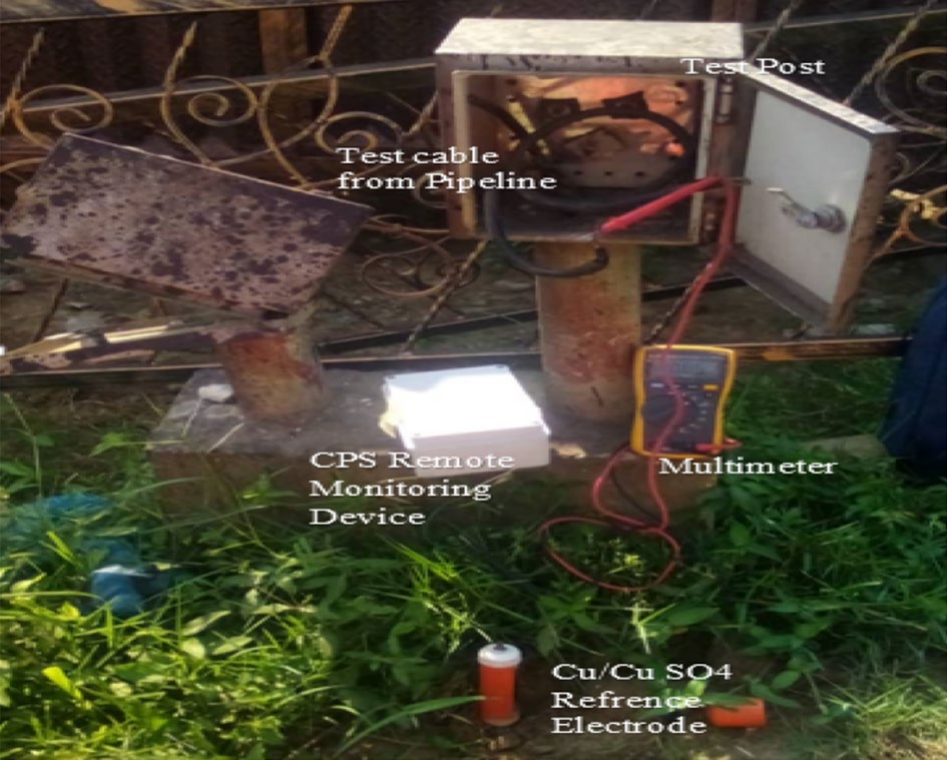
Setup for testing the developed system.

The procedure for testing the developed system is depicted in Fig. 3. During the test, a reference electrode made of copper and copper sulphate (Cu/CuSO4) is inserted into previously moist soil to take measurements from the underground pipeline. The cable from the reference electrode is linked to the voltmeter’s negative terminal, and the cable from the test point for pipeline monitoring is attached to the voltmeter’s positive terminal. The PSP measurement is shown on the voltmeter as reading in volt or milli-volt negative terms relative to the reference electrode. By connecting the two terminals of the device to the reference electrode and the cable from the pipeline, respectively, the developed system measures the PSP data. The readings are transmitted remotely using an IoT platform and are monitored in real-time on the display screen or through a VR environment. The device is tested by switching on the remote monitoring device to transmit data to the Thingspeak IoT server, and then running the application on a phone, PC browser, or VR headset while connected to the VR device. The methods were done according to a standardised protocol as stipulated by the NACE SP0169 standard, and the approval from the institutional ethics committee of Nnamdi Azikiwe University, Awka, Nigeria.

By comparing the measured data from the multimeter with that obtained remotely using the developed device, validation is accomplished. The two measurements are shown on the same plots for analysis, and the correlation coefficient is computed using MATLAB.

A total of two young and healthy participants, comprising one CP technician and one operator, volunteered to participate in the test, and the two participants were briefly trained on the tasks they were to perform. Inclusion criteria for selecting the participants include male or female participants that can read and understand numbers and graphs. Exclusion criteria include participants with visual impairment. These inclusion/exclusion criteria were chosen because the tasks involve reading and interpretation of numerical data and graphs and only healthy and learned participants are required to participate. They were given a consent form to sign before the test. The forms were meant to inform the participants on what to expect during the test, the aim and objectives of the study, as well as intimate them of their rights to either withdraw or continue their participation in the study.

**Figure 4:**
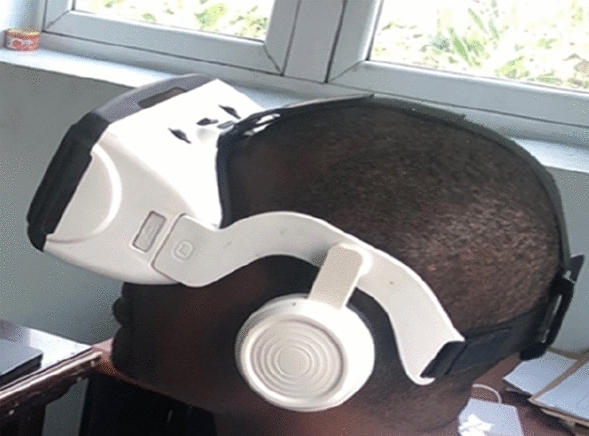
Operator monitoring the PSP readings in the virtual environment using a VR glass headset.

The CP technician in the field manually records the PSP reading from the multimeter every 5 min after setting up the developed system at the test post location. An operator monitors the readings at the same time while wearing the VR headset as depicted in Fig. 4. The virtual environment is launched in the browser to view the PSP reading in real-time on the computer. The ThingSpeak IoT server is also activated to record and store the PSP data update.

### Accordance statement

All methods were carried out by relevant guidelines and regulations. Informed consent was obtained from all subjects prior to the experiments. The study procedure was reviewed and approved by the Ministry of Health, Anambra State of Nigeria, with reference number MH/AWK/M/321/497.

## Results and discussion

**Figure 5:**
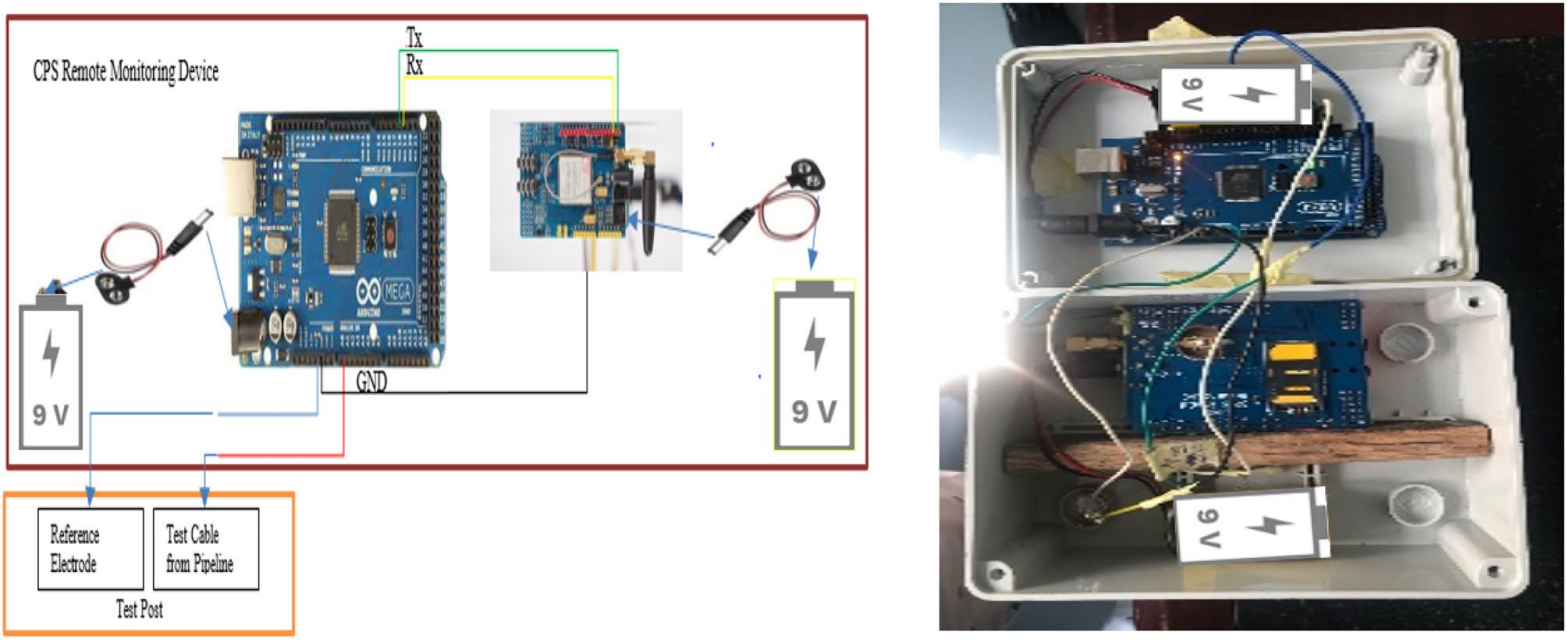
Assembling of the CPS remote monitoring device.

The hardware component of the remote monitoring device was developed using the methods and steps described in “Design of the hardware component of the CPS remote monitoring device” section of this paper and depicted in Figs. 1 and 2. The final assembly is shown in Fig. 5.

To produce an API key and channel id for transmitting the PSP reading from the developed device to the server and retrieve PSP data from the server to the virtual environment, an account was created in the ThingSpeak IoT server. Both while writing and when reading, the channel ID is referred to. PSP reading was chosen as the name of the channel. A USB cable was then used to connect the device to a computer for coding with the Arduino software.

**Figure 6:**
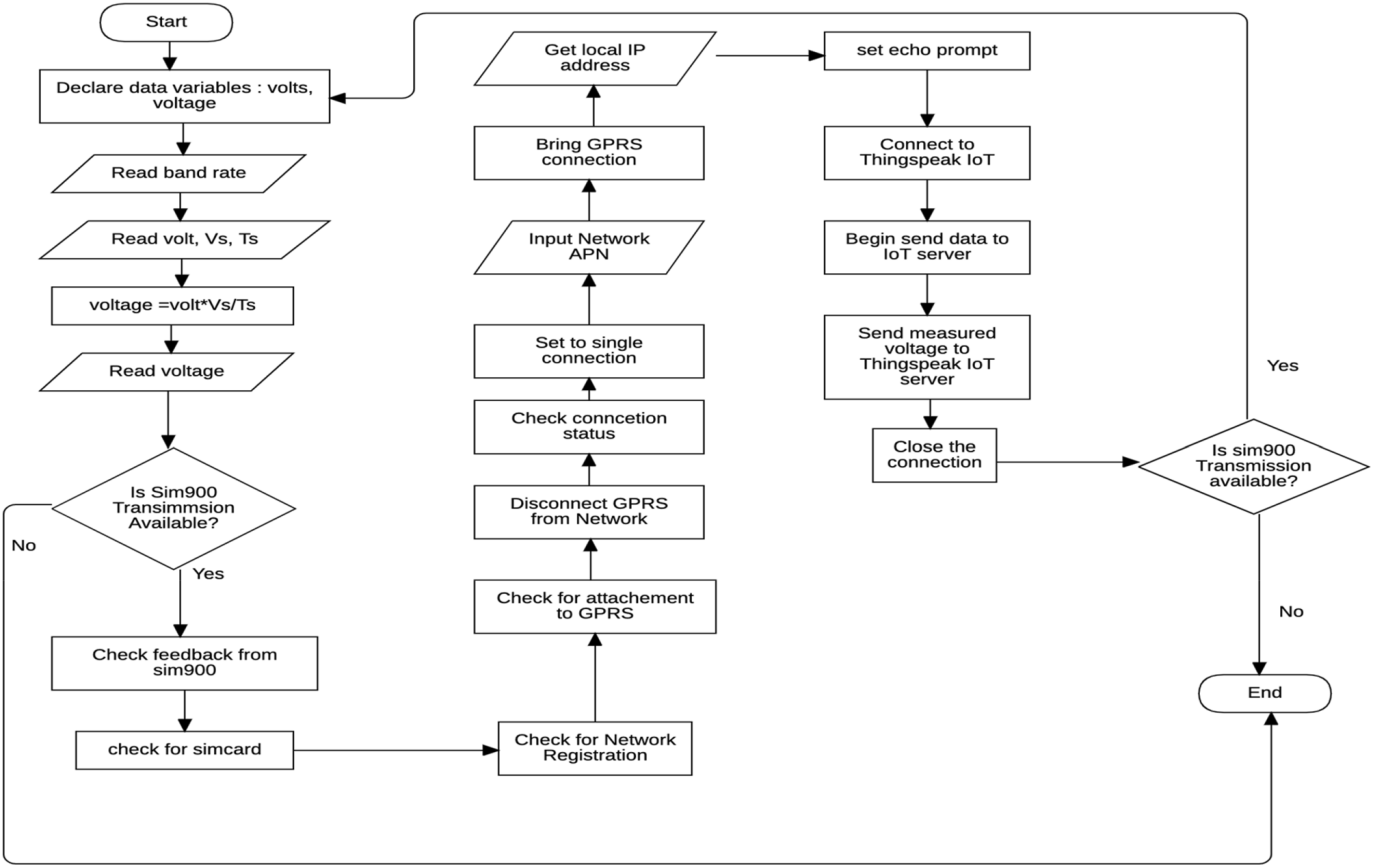
Flowchart developing the software component of the remote monitoring device.

The flowchart depicted in Fig. 6 represents the development process of the software component of the system.

**Figure 7:**
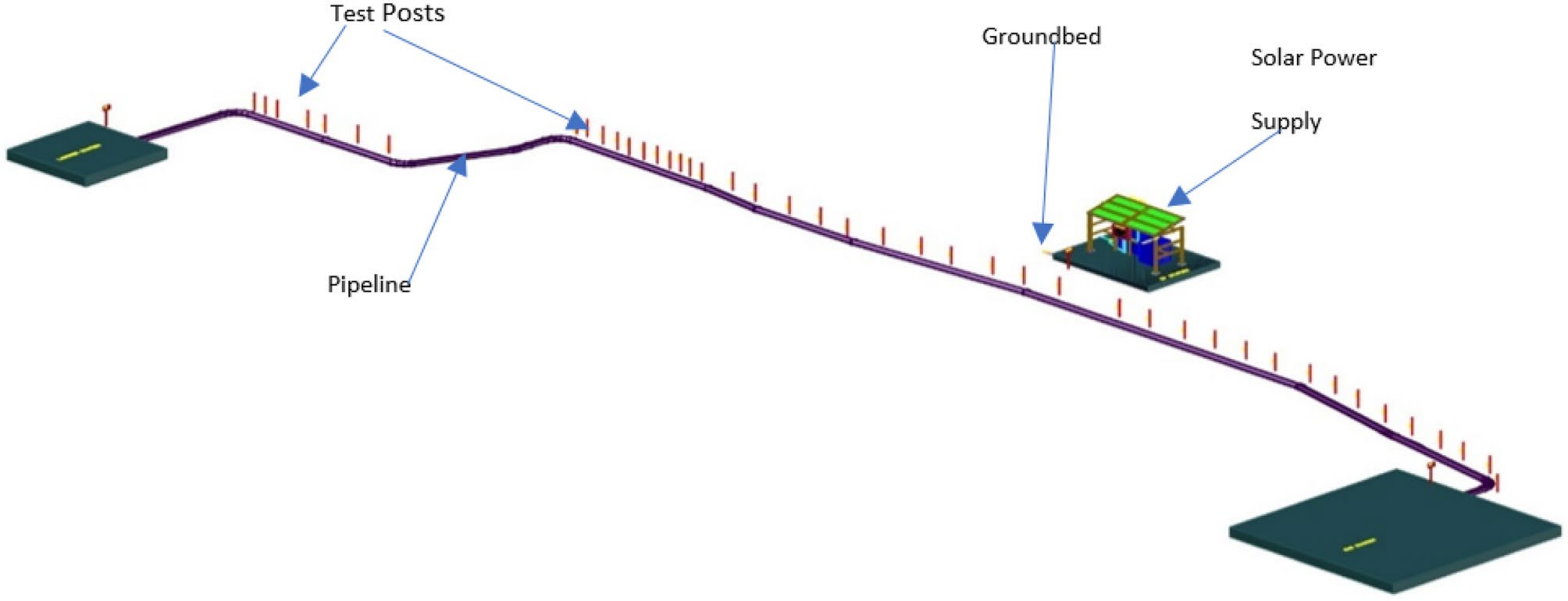
CAD representation of CPS.

After developing the software component, the CAD representation of the CPS was created using AUTOCAD as described in “Validation of the developed system” section and shown in Fig. 7.

**Figure 8:**
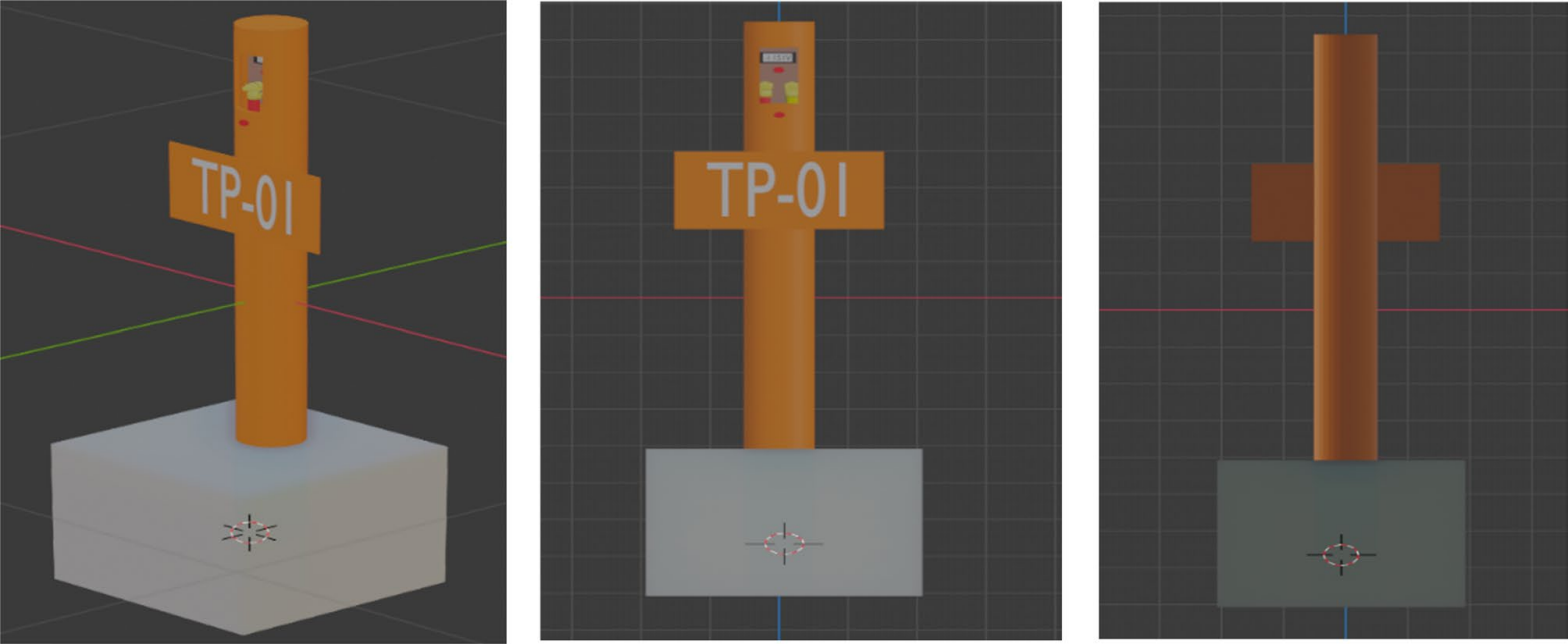
Modelled test posts.

Using the Blender 3D modelling tool, the test post that would be visible when travelling in the virtual environment to track the PSP reading was modelled as shown in Fig. 8.

As seen in Fig. 8, the modelled test posts have two cables. A yellow cable comes from the reference electrode, and a red cable is a test wire from the pipeline. Two cables from the developed device are used to connect to the yellow and red cables. The little display screen on the test post shows the data that has been selected from the IoT server.

**Figure 9:**
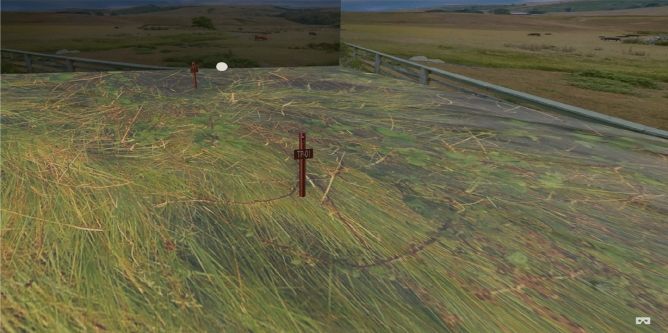
Simulated environment with a test post.

After modelling the test posts and their components using the Blender software, the virtual environment was modelled using the AWS Sumerian. The modelled data from the Blender software are imported into the AWS Sumerian platform and simulated using the Java Script, State machine and the Animation features. Figure 9 depicts the simulated environment.

**Figure 10:**
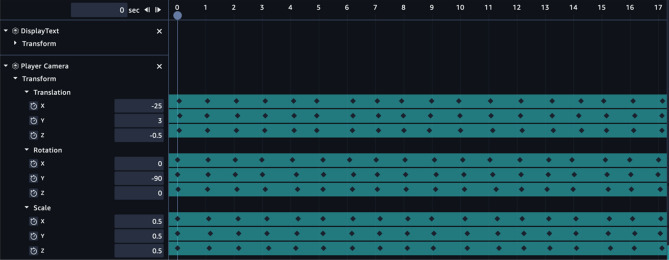
Timeline configuration of immersive movement in AWS Sumerian.

The immersive effect, which will give the user a sense of movement when monitoring the PSP readings in the virtual world using the VR headset, was created using the timeline function of the AWS Sumerian. This is represented in Fig. 10.

The player camera, which shows the view while monitoring in the virtual world, is set up to move locations once per second, as shown in Fig. 10. It was also rotated at the spots to provide the user with several moving views. The first 17 s of the timeline’s snapshot show how the camera moves about. The x, y, and z values for translation, rotation, and scaling are supplied once every second.

When utilising a VR headset, the immersive experience is the result of the timeline setting. This functionality enables people with little or no knowledge of CP to easily monitor the protection status of the CPS on pipelines.

**Figure 11:**
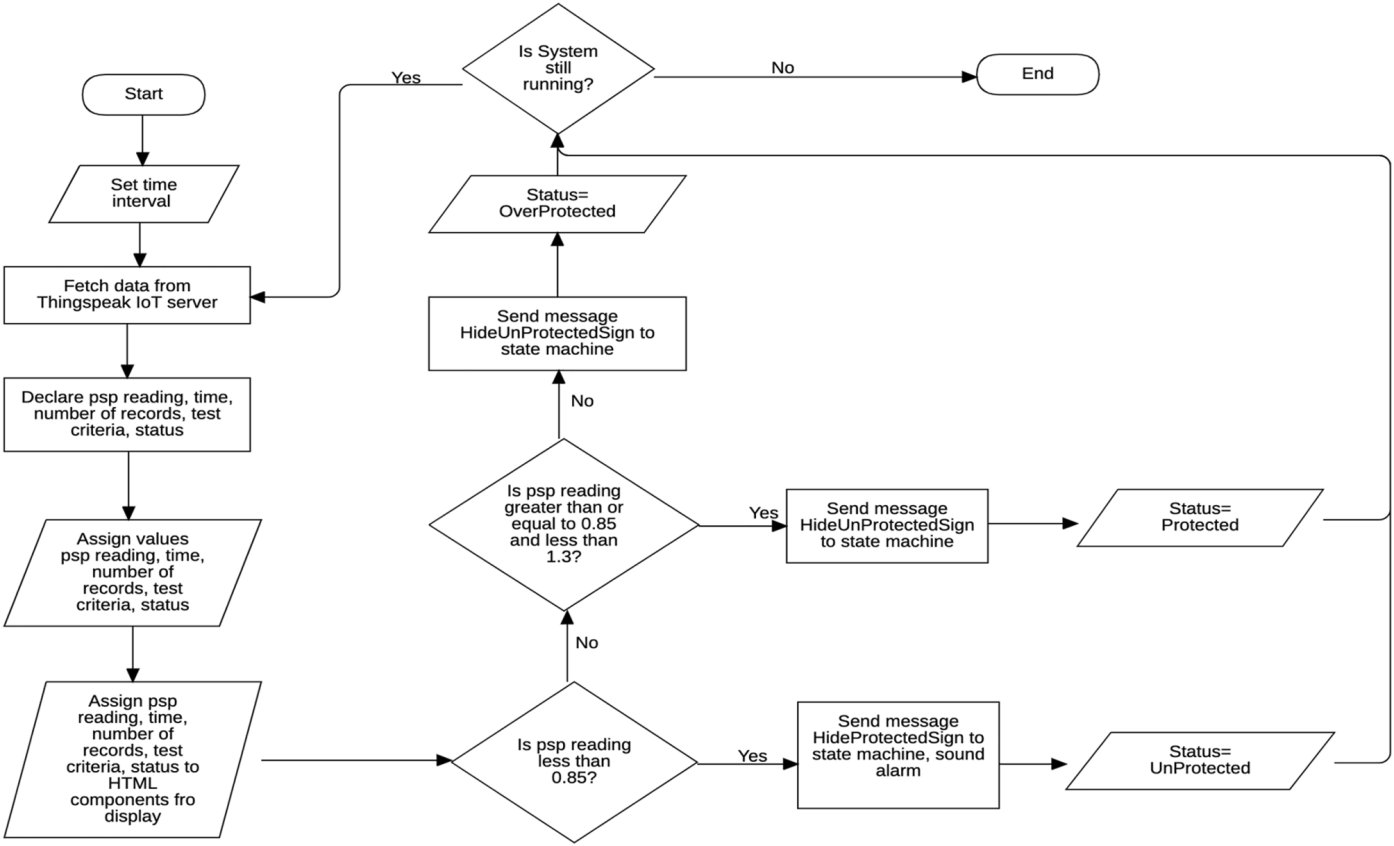
Flowchart for synchronisation.

Through the server, the virtual environment is synchronised with the real-time PSP reading from the developed device. The state machines and scripts of the AWS Sumerian are used to synchronise the virtual environment so the PSP readings can be displayed in real-time as they are measured by the developed device. The lines of codes written to achieve this is summarised in the flow chart of Fig. 11.

**Figure 12:**
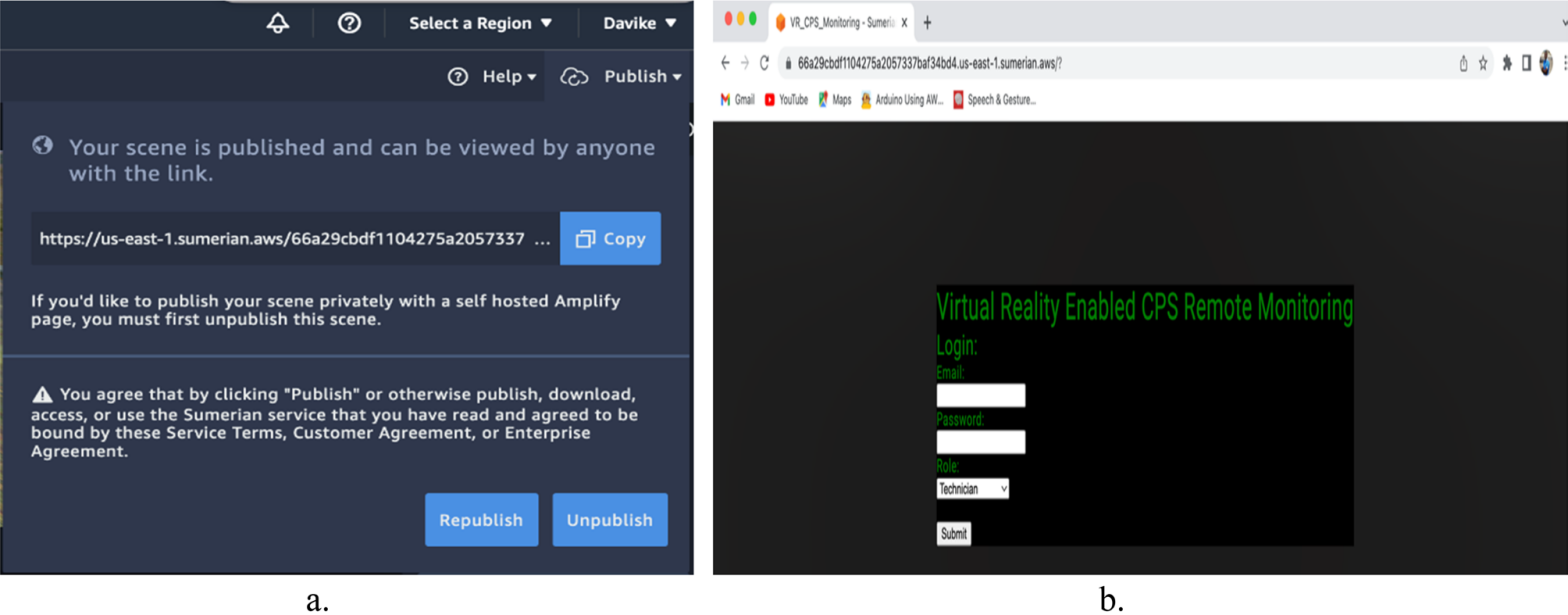
The publishing and browser views in the virtual environment. (**a**) Deployment of the virtual environment. (**b**) Accessing the deployed virtual environment through a web browser.

The virtual environment was distributed to the AWS Sumerian cloud computing platform so that it could be accessible through browsers after being developed and synchronised to provide real-time PSP data. This can be accessed using the link created on a web browser. Figure 12a,b shows the publishing and browser views respectively. If a VR headset is attached to either a mobile device or a computer browser that can access it as it is deployed, the user will have an immersive experience while performing remote CPS monitoring.

### Result of testing the developed system

**Figure 13:**
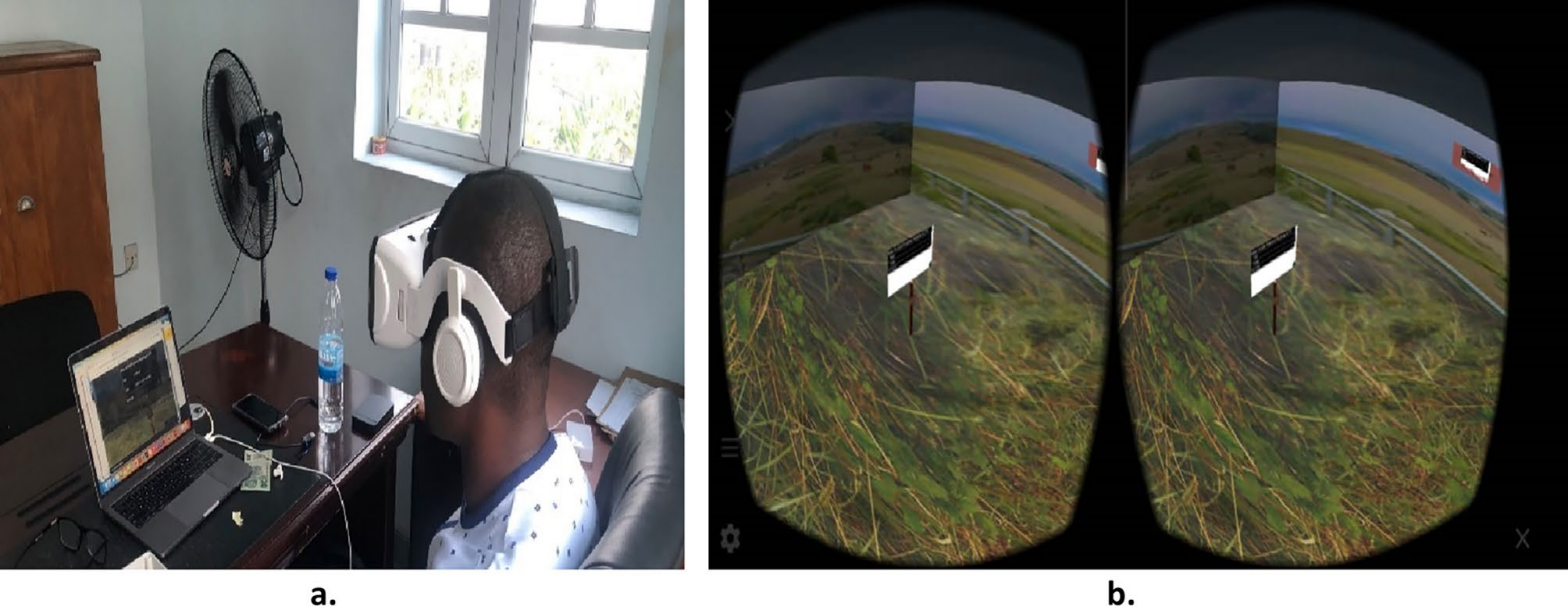
Viewing the feedback of the developed system at a remote location. (**a**) Operator testing the feedback of the developed system at a remote location. (**b**) CPS remote monitoring viewed using a VR glass headset.

The test was carried out as highlighted in “Deployment” section and depicted in Fig. 3, at the designated oil and gas facility of ICSCL, Port Harcourt, Nigeria. During the test, the PSP readings measured by the Multimeter were recorded, while a computer, connected to the internet, was set up so that the PSP readings obtained in the field using the developed hardware component could be monitored by accessing the virtual environment in the browser. An identical webpage is also opened in the mobile browser and a mobile phone is placed into the VR headset glass after logging into the operator’s page and tapping the VR mode icon to log into the VR mode. This resulting view is shown in Fig. 13.

**Figure 14:**
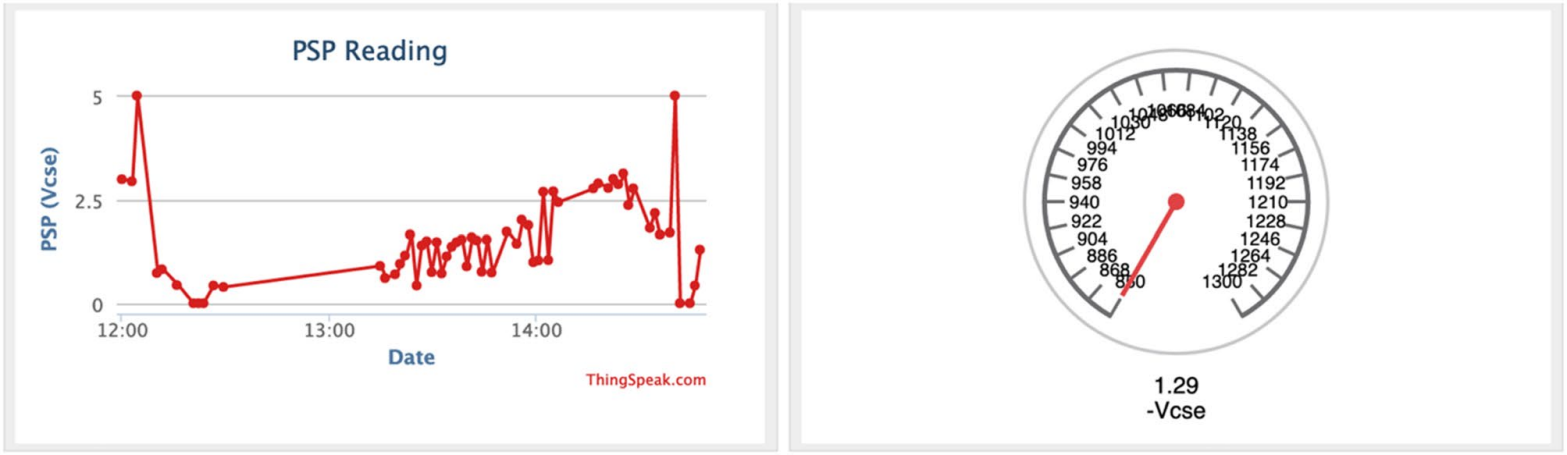
Data captured by the system and transmitted to the ThingSpeak IoT server.

The developed system is first tested for consistency of measurement by plotting its PSP reading against the time taken to capture the data as shown in Fig. 14. The result shows that the PSP values were recorded and transmitted to the IoT server accordingly thereby demonstrating the device's capability to continuously capture PSP data and upload them to the IoT server.

**Figure 15:**
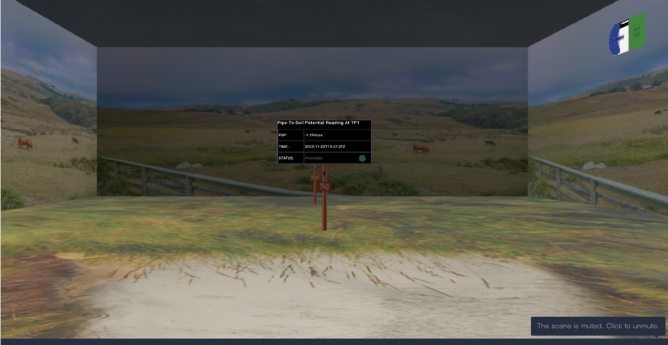
CPS remote monitoring display with protected status.

Figure 15 shows the virtual environment where the PSP reading is displayed to the viewer. A PSP value of − 1.29 Vcse, which is less than the top limit of − 1.3 Vcse and more than the lower limit of − 0.850 Vcse displayed “Protected” with a green sign, indicating that the CPS on the pipeline is protected against corrosion.

**Figure 16:**
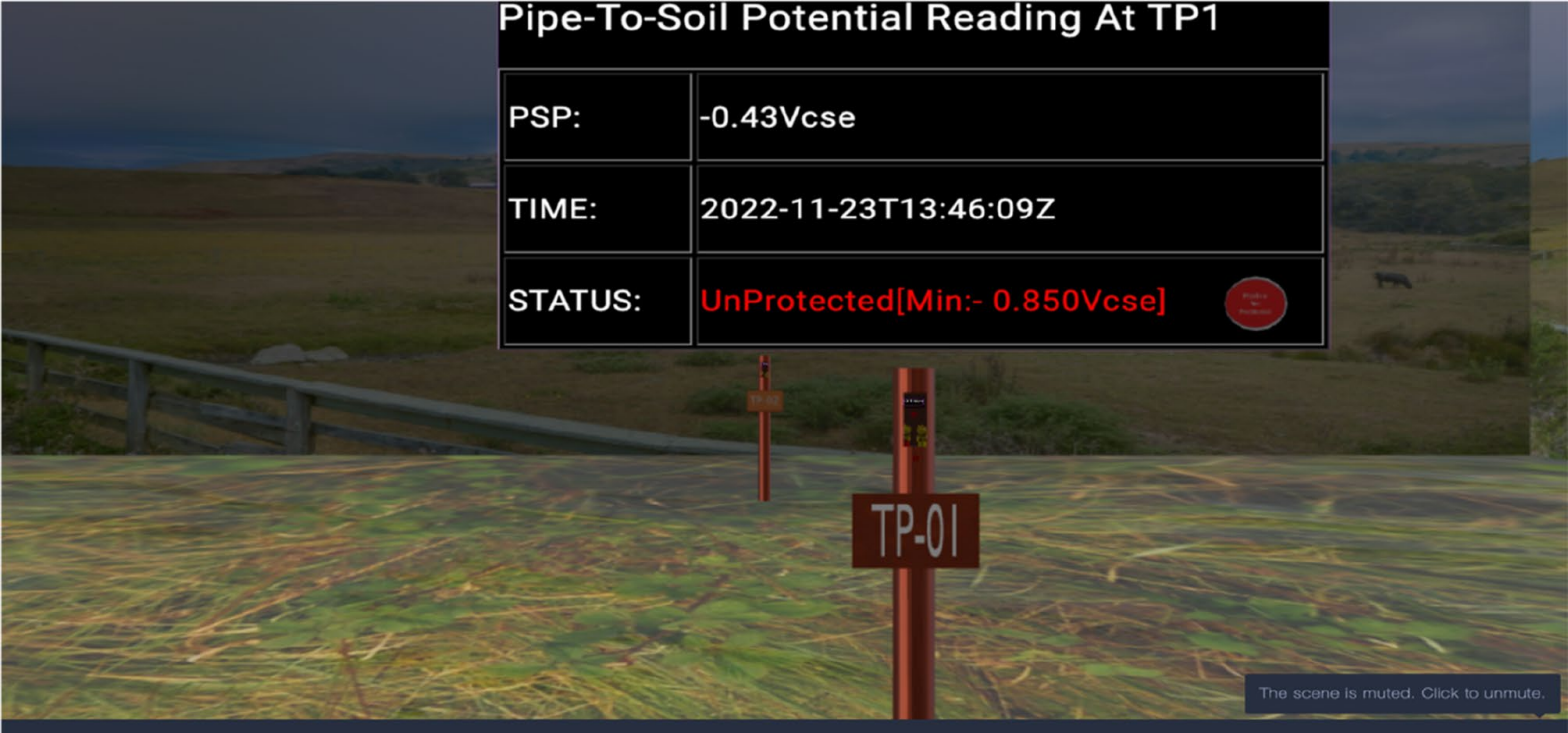
CPS remote monitoring display with unprotected status.

Figure 16 shows a PSP measurement of − 0.43 Vcse, which is less than the limit of − 0.850 Vcse with a visual display of “Unprotected”, a red indicator and an audio alarm. This informs the user that the pipeline is not protected against corrosion.

**Figure 17:**
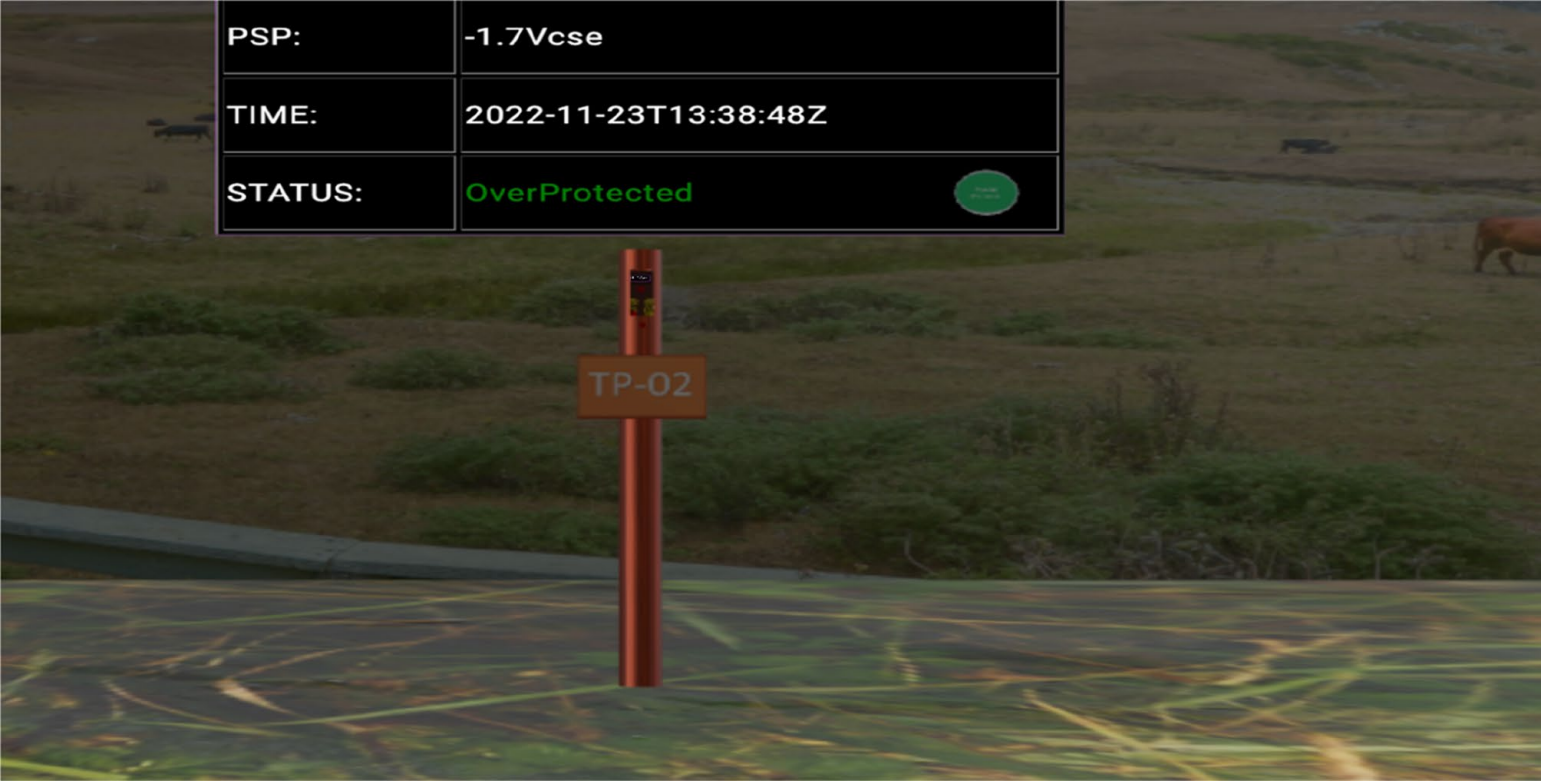
CPS remote monitoring display with over-protected status.

Figure 17 shows a PSP reading of − 1.7 Vcse which is lower than the upper limit of − 1.3 Vcse, and a visual display of “overprotected” status and a green light indication. This informs the user that while the pipeline is protected at the moment, there is a future risk of coating disbandment. This capability is useful since it will provide the user with a clear understanding of how to prepare for predictive maintenance.

The system is developed for use by all categories of users including novice users without prior knowledge of CP systems and monitoring processes for ease of pipeline CPS inspection and maintenance at minimised cost.

### Validation of the developed system

As described in “Deployment” section, the developed system was validated using the multimeter. The Spearman rank was employed to statistically determine the relationship between the measured data from the two devices, as well as the consistency of the measured data using the developed system.

Spearman rank’s correlation coefficient is calculated using the expression:

1 $$rho=1-(6\sum {(d}^{2}))/\left(n\left({n}^{2}-1\right)\right),$$

where rho = Spearman rank correlation coefficient, d is the difference between the ranks of the two variables for each data point, n is the number of data points, and $\sum ({d}^{2})$ is the sum of the squares of the differences between the ranks.

**Figure 18:**
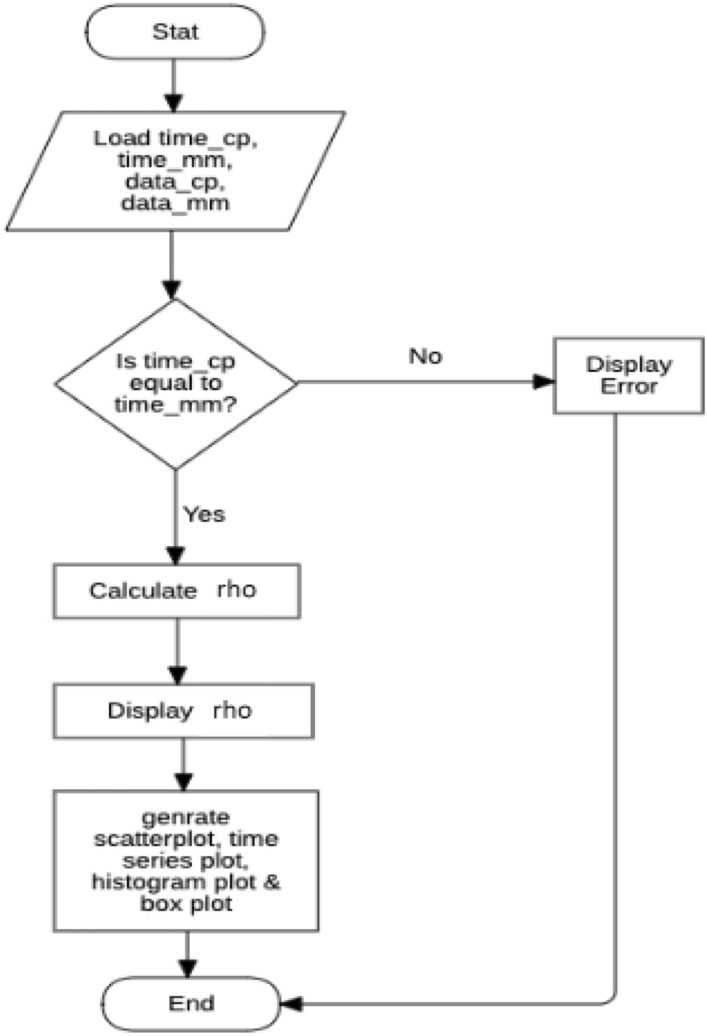
Flowchart for statistical analysis of developed system and multimeter results.

MATLAB was employed for the computation and the result is summarised in the flowchart of Fig. 18.

**Figure 19:**
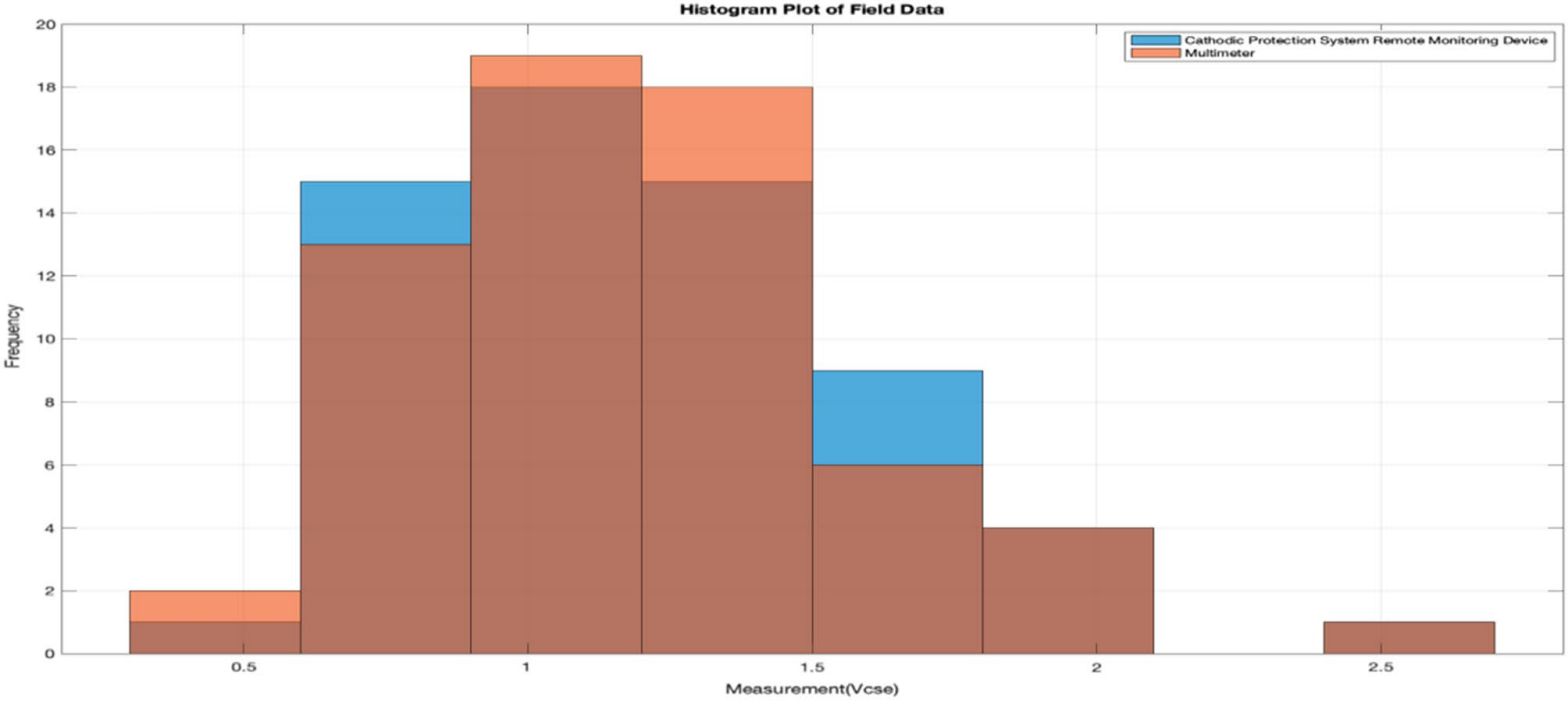
Histogram plot of validation data in MATLAB.

Figure 19 shows the analysis of the closeness of association between the PSP readings captured by the developed system and the multimeter using MATLAB. The Spearman rank’s correlation coefficient obtained is rho = 0.998944 which is very close to 1, indicating that there is a strong correlation between the PSP measurements with the developed system and PSP measurements with the multimeter.

## Conclusion

This research aims to develop an intelligent system that consists of a CPS remote monitoring device, which captures the pipe-to-soil potential (PSP) reading, transmits the measured data to an IoT server, and a virtual environment for analysis and feedback while offering an immersive experience to the user, all in real-time.

A GSM Sim900 Shield and an Arduino Mega 2560 board, which are programmed using the Arduino software, were used to develop the system’s hardware component. The state machines, script, and timeline on the AWS Sumerian platform, the ThingSpeak IoT server, Blender modelling tools for modelling the test posts, and MATLAB for validation were used in the development of the system’s software component. The system demonstrated the consistency of data measurement, with real-time feedback to users when it was installed on actual test posts of an oil and gas facility.

To provide the necessary conditions of safety, economy, and functionality throughout the life cycle of infrastructures such as pipelines, real-time maintenance against corrosion is crucial. This research is aimed at developing a VR-enabled intelligent system that consists of a CPS remote monitoring device which captures the PSP readings on the test posts of oil and gas pipelines, transmits the measured data to an IoT server, and a virtual environment for analysis and feedback while offering an immersive experience to the user, all in real-time. The developed system consists of the hardware component which comprises Sensors, GSM Sim900 Shield and an Arduino Mega 2560 board, programmed using the Arduino software. The state machines, script, and timeline on the AWS Sumerian platform, the ThingSpeak IoT server, Blender modelling tools and AutoCAD software were used to develop the software component of the system. MATLAB was used for validation.

The system also has a virtual reality capability that allows users to observe the pipelines and keep an eye on the PSP readings in real-time, enhancing the user experience.

When tested on actual test posts of oil and gas facilities, the developed system demonstrated the consistency of data measurement and effective monitoring capabilities, with real-time feedback to users, when it was installed on actual test posts of an oil and gas facility. The system’s VR capability allows users to observe the pipelines and keep an eye on the PSP readings in real-time, enhancing the user experience. A comparison of the system's measured data with the traditional method using the multimeter revealed correlation coefficients with a Spearman rank value of rho = 0.998944, indicating the existence of a fully positive association and consistency of measurement.

Further work is recommended to test and validate the device using a wider range of field conditions to ensure its performance and reliability under different environments, weather conditions and soil types. This includes testing in different geographical locations, temperatures, and humidity levels. This will help ensure the device can be used effectively in a variety of real-world settings. The authors also recommend the optimisation of the VR features of the device to provide a more detailed and intuitive representation of the CPS, and to improve the ease and efficiency of maintenance. This can include adding better 3D animations, interactive simulations, and the use of high-quality VR headsets.

This novel system is beneficial to oil and gas facility owners, the oil industry, and the government as it helps to minimise the rate of occurrence of oil spillage and minimise financial losses due to corrosion. The system is deployed to pipeline segments with inaccessible right of way due to bad terrain, security risks, and stray currents. It has proved to be an excellent predictive tool for CPS maintenance in the oil and gas sector. It has helped to reduce the need for the mobilisation of specialised personnel for on-site monitoring and maintenance, which is very costly and often risky. It can be a valuable tool for engineers, technicians, and other professionals involved in designing, installing, and maintaining cathodic protection system.

## Data Availability

The datasets generated during and/or analysed during the current study are available from the corresponding author upon reasonable request.

## References

[1] Eftekhari A, Baghaei Nejad M, Shahrokh Abadi MH (2023). A simple and feasible detection of pipe/tank-wall thinning and corrosion using piezoceramic transducers. Sens. Imaging.

[2] Komary M (2023). Low-cost technologies used in corrosion monitoring. Sensors.

[3] Imran MMH (2023). Application of artificial intelligence in marine corrosion prediction and detection. J. Mar. Sci. Eng..

[4] Hou, B. *et al.* The cost of corrosion in China. *npj Materials Degradation*. 10.1038/s41529-017-0005-2 (2017).

[5] Obike AI, Uwakwe KJ, Abraham EK, Ikeuba AI, Emori W (2020). Review of the losses and devastation caused by corrosion in the Nigeria oil industry for over 30 years. Int. J. Corros. Scale Inhib..

[6] Karami M (2019). Review of corrosion role in gas pipeline and some methods for preventing it. J. Press. Vessel Technol..

[7] Lee J, Siahpour S, Jia X, Brown P (2022). Introduction to resilient manufacturing systems. Manuf. Lett..

[8] Oghli HM, Akhbari M, Kalaki A, Eskandarzade M (2020). Design and analysis of the cathodic protection system of oil and gas pipelines, using distributed equivalent circuit model. J. Nat. Gas Sci. Eng..

[9] Onuoha DO, Mgbemena CE, Godwin HC, Okeagu FN (2022). Application of industry 4.0 technologies for effective remote monitoring of cathodic protection system of oil and gas pipelines—A systematic review. Int. J. Ind. Prod. Eng..

[10] Mobin M, Zehra S, Mobin M, Zehra S (2023). Corrosion control by cathodic protection. Electrochemical and Analytical Techniques for Sustainable Corrosion Monitoring: Advances, Challenges and Opportunities.

[11] Li W (2023). Development of a distributed MR-IoT method for operations and maintenance of underground pipeline network. Tunn. Undergr. Sp. Technol..

[12] Md Asri NS, Mohamad KA, Alias A, Nordin MS (2022). Development of an arduino based electrical instrumentation unit for a remote monitoring cathodic protection system. Evol. Electr. Electron. Eng..

[13] Smalling R, Kruft E, Webb D, Lyon-House L, Smalling R, Kruft E, Webb D, Lyon-House L (2021). Remote monitoring and compute applications. Techniques for Corrosion Monitoring.

[14] Alyoubi KH, Shitharth S, Manoharan H, Khadidos AO, Khadidos AO (2023). Connotation of the fuzzy logic system in underwater communication systems for navy applications with data indulgence route. Sustain. Comput. Inform. Syst..

[15] Anitha G, Manoharan A, Manoharan H, Ganesan P (2022). A survey of security issues in IIoT and fault identification using predictive analysis in industry 4.0. Int. J. Eng. Trends Technol..

[16] Khan, W. Z., Aalsalem, M. Y., Khan, M. K. & Hossain, S. *A Reliable Internet of Things Based Architecture for Oil and Gas Industry* 705–710 (2017).

[17] Rossouw E, Doorsamy W (2021). Predictive maintenance framework for cathodic protection systems using data analytics. Energies.

[18] Parjane VA, Arjariya T, Gangwar M (2023). Corrosion detection and prediction for underwater pipelines using IoT and machine learning techniques. Int. J. Intell. Syst. Appl. Eng..

[19] Omar MZA, Mohamad KA (2023). Development of a remote monitoring system for voltage measurements in cathodic protection. Evol. Electr. Electron. Eng..

[20] Brenna A, Lazzari L, Pedeferri M, Ormellese M (2016). Monitoring cathodic protection of buried pipeline using a potential probe with an embedded zinc reference electrode. J. Mater. Des..

[21] Zhiping Z, Junbi L, Zhengjun W (2012). Pipeline internal corrosion monitoring system with pitting corrosion monitoring ability. Electron. Meas. Technol..

[22] Saluja, A., Costain, J. & Van der Leden, E. Non intrusive online corrosion monitoring. In *Proc. National Seminar & Exhibition on Non-destructive Evaluation* (2009).

[23] Abate F, Di Caro D, Di Leo G, Pietrosanto A (2019). A networked control system for gas pipeline cathodic protection. IEEE Trans. Instrum. Meas..

[24] Peratta, C., Adey, R., Baynham, J. & Dede, A. *Supporting Integrity Management with a CP Digital Twin* (2021).

[25] Peratta, A., Baynham, J., Adey, R. & Pimenta, G. F. Intelligent remote monitoring system for cathodic protection of transmission pipelines. In *Paper Presented at the Corrosion Conference* (2009).

[26] Irannejad M, Iraninejad M (2014). Remote monitoring of oil pipelines cathodic protection system via GSM and its application to SCADA system. Int. J. Sci. Res..

[27] Harbi JA (2021). Application of SCADA system by using (fuzzy logic controller) on the cathodic protection system for oil pipelines. J. Pet. Res. Stud..

[28] Kara, A., Al Imran, M. A. & Karadag, K. Linear wireless sensor networks for cathodic protection monitoring of pipelines. In *Proc. 2019 International Conference on Mechatronics, Robotics and Systems Engineering, MoRSE 2019* 233–236. 10.1109/MoRSE48060.2019.8998664 (Institute of Electrical and Electronics Engineers Inc., 2019).

[29] Al-Faiz MZ, Mezher LS (2012). Cathodic protection remote monitoring based on wireless sensor network. Sci. Res..

[30] Ramzey H, Badawy M, Elhosseini M, Elbaset A (2023). I2OT-EC: A framework for smart real-time monitoring and controlling crude oil production exploiting IIOT and edge computing. Energies.

[31] Choi S, Jung K, Do Noh S (2015). Virtual reality applications in manufacturing industries: Past research, present findings, and future. Concur. Eng. Res. Appl..

[32] Maheswari R, Gnanamalar SSR, Gomathy V, Sharmila B (2018). Real-time environment simulation through virtual reality. Int. J. Eng. Technol..

[33] Kyung J-W, Yokota M, Leelalerkiet V, Ohtsu M (2005). Virtual reality presentation for nondestructive evaluation of Rebar corrosion in concrete based on inverse BEM. J. Korean Soc. Nondestruct. Test..

[34] Webster, R. D. Corrosion prevention and control training in an immersive virtual learning environment. In *NACE International Corrosion Conference Series* (2014).

[35] Walsh, J. A. & Thomas, B. H. Visualising environmental corrosion in outdoor augmented reality. In *Proc. 12th Australian User Interface Conference* 39–46 (2011).

